# High prevalence of exon-13 variants in USH2A-related retinal dystrophies in Taiwanese population

**DOI:** 10.1186/s13023-024-03238-2

**Published:** 2024-06-15

**Authors:** Yu-Wei Lin, Yu-Shu Huang, Chien-Yu Lin, Chao-Wen Lin, Chen-Chi Wu, Chang-Hao Yang, Chung-May Yang, Pei-Lung Chen, Ta-Ching Chen

**Affiliations:** 1 Department of Medical Education, National Taiwan University Hospital, Taipei, Taiwan; 2 Graduate Institute of Clinical Medicine, College of Medicine, National Taiwan University, Taipei, Taiwan; 3 Department of Ophthalmology, National Taiwan University Hospital, No 7, Chung-Shan S. Rd, Taipei, Taiwan; 4 Graduate Institute of Medical Genomics and Proteomics, Medical College, National Taiwan University, No. 2, Xuzhou Road, 5F. Taipei, Taiwan; 5 Department of Medical Genetics, National Taiwan University Hospital, Taipei, Taiwan; 6 Center of Frontier Medicine, National Taiwan University Hospital, Taipei, Taiwan; 7 Department of Otolaryngology, National Taiwan University Hospital, Taipei, Taiwan

**Keywords:** *USH2A*, Usher syndrome, Retinitis pigmentosa, Exon 13, Gene therapy

## Abstract

**Background:**

Biallelic pathogenic variants in *USH2A* lead to Usher syndrome or non-syndromic retinitis pigmentosa, and shown to have geographical and ethnical distribution in previous studies. This study provided a deeper understanding of the detailed clinical features using multimodal imaging, genetic spectrum, and genotype–phenotype correlations of *USH2A*-related retinal dystrophies in Taiwan.

**Results:**

In our cohort, the mean age at first visit was 47.66 ± 13.54 years, and the mean age at symptom onset, which was referred to the onset of nyctalopia and/or visual field constriction, was 31.21 ± 15.24 years. Among the variants identified, 23 (50%) were missense, 10 (22%) were splicing variants, 8 (17%) were nonsense, and 5 (11%) were frameshift mutations. The most predominant variant was c.2802T>G, which accounted for 21% of patients, and was located in exon 13. Patients with truncated alleles had significantly earlier symptom onset and seemly poorer disease progression regarding visual acuity, ellipsoid zone line length, and hypofluorescent lesions in the macula than those who had the complete gene. However, the clinical presentation revealed similar progression between patients with and without the c.2802T>G variant. During long-term follow-up, the patients had different ellipsoid zone line progression rates and were almost evenly distributed in the fast, moderate, and slow progression subgroups. Although a younger onset age and a smaller baseline intact macular area was observed in the fast progression subgroup, the results showed no significant difference.

**Conclusions:**

This is the first cohort study to provide detailed genetic and longitudinal clinical analyses of patients with *USH2A-*related retinal dystrophies in Taiwan. The mutated allele frequency in exon 13 was high in Taiwan due to the predominant c.2802T>G variant. Moreover, truncated variants greatly impacted disease progression and determined the length of therapeutic windows. These findings provide insight into the characteristics of candidates for future gene therapies.

**Supplementary Information:**

The online version contains supplementary material available at 10.1186/s13023-024-03238-2.

## Background

Retinitis pigmentosa (RP, OMIM: 268000), the most common form of inherited retinal dystrophy (IRD), affects an estimated 1 in 4000 individuals worldwide and severely destroys visual function in young people [1]. RP is characterized by progressive rod cell degeneration, leading to night blindness and visual field loss. Once cone cells are affected later in life, the loss of visual acuity develops. The most common inheritance pattern of RP is autosomal recessive, accounting for 50% to 60% of all patients with this disease, whereas autosomal dominant and X-linked inheritance occur in only 30% to 40% and 5% to 15%, respectively [2]. Currently, over 100 causal genes of autosomal recessive RP (arRP) are registered in the Retinal Information Network (RetNet) database, demonstrating the genetic heterogeneity of the disease [3].

Usher syndrome (USH; OMIM:276900) is an autosomal recessive disorder characterized by RP and hearing loss, with or without vestibular dysfunction. Therefore, the syndrome is clinically classified into three subtypes based on the severity and progression of vision and hearing loss and the presence of balance [4]. USH type 2 (USH2), the most common type, accounting for over 50% of USH syndrome cases, is typically characterized by congenital moderate-to-severe hearing loss and a normal vestibular response. Approximately 80% of USH2 cases are caused by mutations of the *USH2A* gene, which is mapped to chromosome 1q41 and comprises about 15 kb of the DNA coding sequence, containing 72 exons that encode usherin [5–7]. Usherin is a ciliary transmembrane protein that includes laminin epidermal growth factor motifs, a transmembrane domain, and repeats of fibronectin type-III motifs. Usherin is predominantly expressed in the retinal photoreceptors and hair cells in the cochlea [8–11].

Moreover, alterations in *USH2A* levels result in various phenotypes and disease severities [12, 13]. Previous studies have shown that mutations in *USH2A* are responsible for at least 7% of nonsyndromic RP and 57 to 67.7% of USH [1, 14]. To date, among the more than 1,000 pathogenic or likely pathogenic variants reported, two ancestral variants c.2299delG and c.2276G > T are the most frequent in Europe and the United States [15, 16]. The two variants c.2802T>G and c.8559-2A>G were the most reported in a Chinese cohort [17]. However, few studies on *USH2A*-associated retinal dystrophies have been conducted in the Taiwanese population.

The use of gene therapy has skyrocketed in recent decades. Due to the fact that adeno-associated virus (AAV)-based gene augmentation therapies, which the cargo limit is 4.7kb, cannot accommodate the USH2A coding sequence (15kb), antisense oligonucleotide (ASO)-based therapies are gaining popularity [18, 19]. Previous study [20] had demonstrated that skipping of exon 13 could be achieved at the mRNA level by ASOs and resulted in a shorter usherin with residual function. Therefore, understanding the genetic variants and genotype–phenotype correlation is important for bridging future therapies that provide hope to patients with no available therapeutic options. In this study, we aimed to investigate the detailed clinical features, genetic spectrum, and genotype–phenotype correlations of *USH2A*-related retinal dystrophies in Taiwan.

## Results

### Demographics

A total of 41 subjects with two alleles of *USH2A* variants were enrolled in our study; 19 (46%) were male, and 22 (54%) were female. The mean age at the first visit was 47.66 (SD ± 13.54) years. The mean age at symptom onset, which was referred specially as the age that the patients reported nyctalopia and/or visual field constriction, was 31.21 (SD ± 15.24) years. Hearing impairment was reported in 15 (37%) patients. The mean follow-up period, defined by the length of time between the first and last visits, was 46.89 (SD ± 28.31) months. Detailed clinical presentations and genotype results are presented in Table 1.

**Table 1:** Clinical presentations and genotypes of the patients with *USH2A*-related retinal dystrophies

Proband	Gender	Age at visit	Onset age	F/U time (months)	Nucleotide change	Visual acuity at first visit (LogMAR)		Visual acuity at a recent visit (LogMAR)		EZ line progression (mm/yr)		Macula lesion area progression (%/yr)		Hearing impairment
						OD	OS	OD	OS	OD	OS	OD	OS	
01	M	41	30	N.A	c.[820C > T];[15178 T > C]	0.52	0.10	N.A	N.A	N.A	N.A	N.A	N.A	No
02	F	29	12	68.03	c.[2187C > A];[14583-40_14583-10del]	0.52	0.52	0.52	0.40	0.172	0.235	3.167	3.241	Yes
03	M	60	50	109.77	c.3665C > T(;)4576G > A	0.52	0.82	0.30	0.30	N.A	N.A	N.A	N.A	No
04	F	29	15	77.43	c.[100_101insT];[10582A > G]	0.40	0.70	0.70	0.82	0.003	0.003	N.A	9.303	No
05	F	39	15	65.13	c.12503C > A(;)13731_13743del	0.40	1.00	0.40	1.52	N.A	N.A	0.541	4.348	No
06	M	55	30	51	c.2802 T > G(;)2955C > A	0.10	0.15	0.22	0.10	0.144	0.176	4.616	N.A	No
07	F	33	15	80.77	c.2802 T > G(;)5581G > A	0.70	0.52	2.00	0.70	0.012	0.023	9.550	8.549	Yes
08	M	73	30	52.9	c.11389 + 3A > T(;)13339A > G	0.70	0.70	1.00	0.82	0.088	0.075	N.A	N.A	No
09	M	46	31	68.17	c.538 T > C(;)2802 T > G	0.70	0.70	0.80	2.00	N.A	N.A	3.437	1.212	No
10	M	22	15	62.13	c.13045_13046insG(;)14287G > A	0.10	0.30	0.00	0.00	0.016	0.016	4.819	3.738	Yes
11	M	62	50	90.87	c.3635C > T(;)4576G > A	0.30	0.52	0.22	0.52	0.014	0.044	1.983	2.552	No
12	F	60	50	110.13	c.2802 T > G(;)10859 T > C	0.70	0.70	2.00	1.30	0.061	0.050	5.062	5.025	No
13	F	50	13	47.47	c.11712-2A > C(;)5603_5613del	0.30	0.22	0.70	0.30	0.051	0.035	1.096	1.248	Yes
14	M	40	41	58.87	c.4384del(;)5329C > T	0.10	0.05	0.10	0.05	0.048	0.033	8.038	10.514	No
15	F	45	21	37.57	c.951del(;)12,066 + 21769_14792-738del*	2.30	2.30	2.30	2.30	N.A	N.A	4.196	N.A	Yes
16	F	42	35	N.A	c.4576G > A(;)9570 + 1G > A	1.00	1.00	N.A	N.A	N.A	N.A	N.A	N.A	Yes
17	F	30	21	37.2	c.5836C > T(;)10859 T > C	0.22	0.22	0.15	0.22	0.210	0.210	0.813	N.A	No
18	F	35	25	6.17	c.5836C > T(;)10859 T > C	0.05	0.05	0.05	0.05	N.A	N.A	N.A	N.A	No
19	F	51	21	10.8	c.6485 + 5G > T(;)6806-5434_6658-1443del*	1.70	1.52	1.40	1.30	N.A	N.A	N.A	N.A	Yes
20	F	46	40	37.77	c.2802 T > G(;)13007G > A	0.05	0.15	0.00	0.05	0.020	0.020	2.832	2.190	No
21	F	55	8	20.43	c.2802 T > G(;)13007G > A	0.52	0.70	0.70	0.70	0.153	0.112	5.945	8.452	No
22	M	68	65	36.67	c.2802 T > G(;)13465G > A	0.30	0.52	0.22	0.30	0.062	0.147	3.877	1.939	No
23	F	54	46	35.03	c.13339A > G(;)14,134-11 T > C	0.70	0.70	0.70	0.70	0.021	0.045	5.436	7.190	No
24	F	46	35	N.A	c.2802 T > G(;)[7097C > T;7095delC]	1.30	1.30	N.A	N.A	N.A	N.A	N.A	N.A	No
25	F	75	45	26.43	c.2802 T > G(;)15178 T > C	0.52	0.70	0.70	0.52	0.086	0.059	2.045	3.225	No
26	M	48	15	14.03	c.13049_13062del(;)4251 + 185_652-12340del*	1.52	1.40	1.30	1.30	N.A	N.A	2.420	2.614	No
27	F	28	28	N.A	c.2802 T > G(;)9041C > A	0.00	0.00	N.A	N.A	N.A	N.A	N.A	N.A	No
28	F	55	50	28.17	c.2802 T > G(;)4576G > A	0.80	1.52	2.00	2.00	N.A	N.A	2.680	1.268	Yes
29	M	62	41	28.97	c.4030A > G(;)13483C > T	0.22	0.10	0.30	0.22	0.079	0.108	4.329	5.957	No
30	M	58	40	22.73	c.2802 T > G(;)9469C > T	0.15	1.30	0.70	2.30	N.A	N.A	2.076	1.395	Yes
31	M	32	28	37.67	c.8232G > C(;)2810-3265_2993 + 3480del*	0.22	0.15	0.22	0.22	0.099	0.072	2.599	1.719	Yes
32	F	63	40	N.A	c.8559-2A > G(;)2187C > A	2.80	2.80	2.90	2.90	N.A	N.A	N.A	N.A	Yes
33	F	53	4	N.A	c.4576G > A(;)484A > G	1.30	1.52	N.A	N.A	N.A	N.A	N.A	N.A	Yes
34	M	59	32	N.A	c.5299-2A > G(;)11333C > T	1.00	1.30	N.A	N.A	N.A	N.A	N.A	N.A	No
35	M	34	15	N.A	c.4987 + 1G > A(;)8232G > C	0.10	0.22	N.A	N.A	N.A	N.A	N.A	N.A	Yes
36	F	35	12	8.33	c.2802 T > G(;)14287G > A	0.15	0.22	0.22	0.40	N.A	N.A	N.A	N.A	No
37	M	47	18	N.A	c.2187C > A(;)9570 + 1G > A	2.30	0.80	N.A	N.A	N.A	N.A	N.A	N.A	Yes
38	M	28	15	N.A	c.5572 + 1G > A(;)10861A > T	0.22	0.10	N.A	N.A	N.A	N.A	N.A	N.A	Yes
39	M	44	41	N.A	c.9469C > T(;)13339A > G	0.10	0.15	N.A	N.A	N.A	N.A	N.A	N.A	No
40	M	68	57	29.13	c.15178 T > C; c.13339A > G; c.2653C > T	0.30	0.22	0.22	0.30	0.140	0.087	0.515	2.632	No
41	F	54	52	N.A	c.13339A > G; c.4576G > A	0.22	0.10	N.A	N.A	N.A	N.A	N.A	N.A	No

*AR* autosomal recessive, * with structural variants, *N.A* not available, *OD* oculus dexterous, *OS* oculus sinister

### Genetic spectrum of *USH2A*-related retinal dystrophies in Taiwanese

**Fig. 1:**
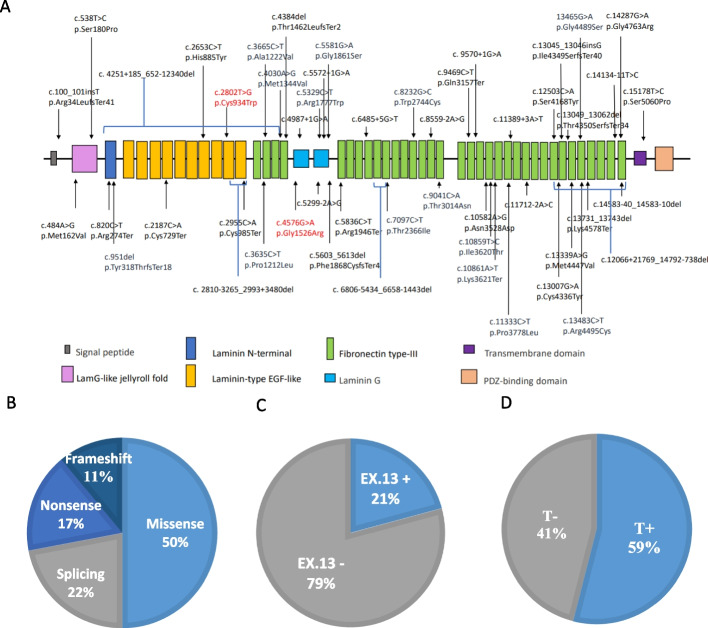
Detailed genetic information in our cohort. **A** Schematic of the USH2A gene structure with the locations of the variants showed in our cohort. The two most predominant variants in our cohort were highlighted in red. **B** Types of mutations in our cohort. **C** Percentage of subjects with exon 13 (EX.13 +) and others (EX.13-). **D** Percentage of subjects with/without the existence of truncated alleles. Group T-: without truncated alleles; Group T + : with at least one truncated allele

Capture-based NGS was performed for all recruited patients to identify 46 variants distributed throughout the entire *USH2A* gene sequence (Fig. 1A); four subjects had structural variants (Table 1). *USH2A* variants were confirmed according to American College of Medical Genetics and Genomics guidelines. Regarding the types of the variants, 23 (50%) were missense, 10 (22%) were splicing variants, 8 (17%) were nonsense, and 5 (11%) were frameshift mutations (Fig. 1B). The “splicing variant” was defined in our cohort as the variant located in the intronic regions which resulted in RNA splicing defect and was considered as a truncated mutation. The two most predominant variants in this study were c.2802T>G (p.Cys934Trp) and c.4576G>A (p.Gly1526Arg) (Table 2), with relative allele frequencies of 15.85% and 7.32%, respectively. The most common position of the variants was exon 13, with a frequency of 21% (Fig. 1C). The most common variant domain documented in this study was the fibronectin type-III domain, with 63% (*n* = 29). Seven variants were in the laminin G domain, four were in the laminin-type epidermal growth factor-like domain, and the remaining were in the laminin G-like jelly roll fold, laminin N-terminal, and transmembrane domains.

**Table 2:** Results of the genetic analysis of the USH2A variants in our cohort

Variant (nucleotide change; amino acid change)	Variant type	Allele count	Position	Relative allele frequency (%)	ACMG classification
c.2802 T > G (p.Cys934Trp)	M	13	ex13	15.85	P
c.4576G > A (p.Gly1526Arg)	M	6	ex21	7.32	LP
c.13339A > G (p.Met4447Val)	M	5	ex63	6.10	LP
c.2187C > A (p.Cys729Ter)	N	3	ex13	3.66	P
c.5836C > T (p.Arg1946Ter)	N	2	ex29	2.44	P
c.8232G > C (p.Trp2744Cys)	M	2	ex42	2.44	LP
c.9469C > T (p.Gln3157Ter)	N	2	ex48	2.44	P
c.10859 T > C (p.Ile3620Thr)	M	2	ex55	2.44	LP
c.14287G > A (p.Gly4763Arg)	M	2	ex65	2.44	P
c.15178 T > C (p.Ser5060Pro)	M	2	ex70	2.44	VUS
c.3665C > T (p.Ala1222Val)	M	1	ex17	1.22	LP
c.8559-2A > G	S	1	in42	1.22	P
c.9041C > A (p.Thr3014Asn)	M	1	ex45	1.22	VUS
c.100_101insT (p.Arg34LeufsTer41)	F	1	ex2	1.22	P
c.484A > G(p.Met162Val)	M	1	ex2	1.22	LP
c.538 T > C (p.Ser180Pro)	M	1	ex3	1.22	LP
c.820C > T (p.Arg274Ter)	N	1	ex5	1.22	P
c.951delG (p.Tyr318ThrfsTer18)	N	1	ex6	1.22	LP
c.2653C > T(p.His885Tyr)	M	1	ex13	1.22	LP
c.2955C > A (p.Cys985Ter)	N	1	ex14	1.22	P
c.3635C > T (p.Pro1212Leu)	M	1	ex17	1.22	LP
c.4030A > G (p.Met1344Val)	M	1	ex18	1.22	VUS
c.4384del (p.Thr1462LeufsTer2)	F	1	ex20	1.22	P
c.4987 + 1G > A	S	1	in24	1.22	P
c.5299-2A > G	S	1	in26	1.22	P
c.5329C > T (p.Arg1777Trp)	M	1	ex27	1.22	LP
c.5572 + 1G > A	S	1	in27	1.22	P
c.5581G > A (p.Gly1861Ser)	M	1	ex28	1.22	LP
c.5603_5613del (p.Phe1868CysfsTer4)	F	1	ex28	1.22	P
c.6485 + 5G > T	S	1	in33	1.22	P
c.7097C > T (p.Thr2366Ile)	M	1	ex37	1.22	LP
c.9570 + 1G > A	S	1	in48	1.22	P
c.10582A > G (p.Asn3528Asp)	M	1	ex53	1.22	LP
c.10861A > T (p.Lys3621Ter)	N	1	ex55	1.22	LP
c.11333C > T (p.Pro3778Leu)	M	1	ex58	1.22	VUS
c.11389 + 3A > T	S	1	in58	1.22	LP
c.11712-2A > C	S	1	in60	1.22	P
c.12503C > A (p.Ser4168Tyr)	M	1	ex63	1.22	LP
c.13007G > A (p.Cys4336Tyr)	M	1	ex63	1.22	LP
c.13045_13046insG (p.Ile4349SerfsTer40)	F	1	ex63	1.22	P
c.13049_13062del (p.Thr4350SerfsTer34)	F	1	ex63	1.22	P
c.13465G > A (p.Gly4489Ser)	M	1	ex63	1.22	LP
c.13483C > T (p.Arg4495Cys)	M	1	ex63	1.22	LP
c.13731_13743del (p.Lys4578Ter)	N	1	ex63	1.22	P
c.14134-11 T > C	S	1	in64	1.22	VUS
c.14583-40_14583-10del	S	1	in66	1.22	VUS

*M* missense, *N* nonsense, *S* splicing variant, which located in the intronic regions that lead to RNA splicing decfect, *F* frameshift mutation, *ACMG* American College of Medical Genetics and Genomics, *P* pathogenic, *LP* likely pathogenic, *VUS* variant of uncertain significance, *ex* exon, *in* intron

### Clinical presentations of *USH2A*-related retinal dystrophies based on the existence of truncated alleles

To investigate the correlation between the existence of truncated alleles and clinical presentation, such as age at symptom onset, age at the first visit, visual acuity at the first and recent visits, EZ line progression, and fundus autofluorescence (FAF) lesion progression, a detailed comparison was performed among groups T- (those with no truncated variants) and T+ (those with at least one truncated variant), which comprised 17 (41%) and 24 (59%) patients, respectively (Fig. 1D).

The ages at symptom onset and first visit were much lower in group T+ (mean = 26.55 and 43.67 years, respectively) than in group T- (mean = 37.24 and 53.29 years, respectively). The differences between the age at symptom onset and at the first visit in these two groups were statistically significant (*p* = 0.02 and 0.03, respectively).

**Fig. 2:**
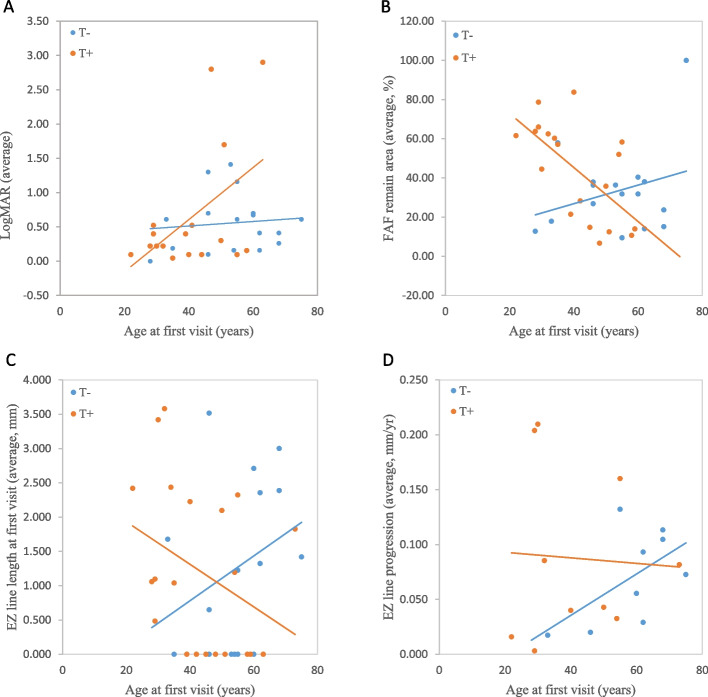
Comparison of the clinical progression of patients with/without truncated alleles in our cohort. Every point represents an individual patient. The blue and orange lines are the linear regression lines for group T- (without truncated alleles) and T + (with at least one truncated allele), respectively. OD, right eye; OS, left eye. **A** LogMAR of the average of both eyes at the first visit, presented as a function of age. **B** The average intact area of macula of both eyes on fundus autofluorescence (FAF, %) at the first visit, presented as a function of age. **C** EZ line length of the average of both eyes at the first visit, presented as a function of age. **D** EZ line loss progression rates (mm/year) of the average of both eyes, presented as a function of age

Visual acuity (average of both eyes, calculated as logarithm of the minimum angle of resolution, logMAR) at the first visit was 0.77 in group T+; whereas, that in group T- was 0.56. At a recent visit, the visual acuities (average of both eyes) were 0.83 for group T+ and 0.73 for group T-. Figure 2A showed the baseline average visual acuities of both eyes. The visual acuity progression seemed to be faster in group T+ than in group T- along with increasing age.

Figure 2B showed the average preserved areas of the macula at the first visit of both eyes. The mean baseline intact areas of the macula were 42.45% in group T+ and 34.17% in group T-. The decrease in the intact macular area was faster in group T+ with increasing age. As for the hypofluorescent area progression rate at the macula, the average rates were 3.88 %/year in group T+ and 3.76 %/year in group T-, respectively.

Figure 2C showed the patients’ average EZ line lengths of both eyes plotted at the first visit. The mean baseline EZ line length was 1.218 mm in group T+, while in group T-, it measured 1.267 mm. The decrease in the EZ line length was faster in group T+ with increasing age. As for the analysis of the EZ line loss progression rate at the fovea, the average rates were 0.088 mm/year in group T+ and 0.071 mm/year in group T-, respectively. In the linear model showing the progression rate of EZ line loss as a function of age (Fig. 2D), group T- demonstrated faster progression rate along with increasing age; whereas, group T+ showed a decreasing progression rate along with age.

More patients in group T+ reported hearing impairment. Twelve (50%) in group T+ suffered from hearing loss, and twelve (50%) did not; whereas, three (18%) patients in group T- had hearing loss, and fourteen (82%) patients did not. Differences were analyzed using Pearson's chi-square test and showed statistical significance (*p* = 0.034).

### Clinical presentations of *USH2A*-related retinal dystrophies based on the existence of C.2802T>G Variant

c.2802T>G (p.Cys934Trp), being the most predominant variant in our study, accounted for 15.85% (13/82); i.e., 32% (*n* = 13) of subjects had c.2802T>G variant, and 68% (*n* = 28) did not. The ages at symptom onset and the first visit were higher in patients with the c.2802T>G variant (mean = 46.93 and 50.77 years, respectively) than in patients without (mean = 29.54 and 34.54 years, respectively). However, the differences between the age at symptom onset and the age at first visit in these two groups were not statistically significant.

**Fig. 3:**
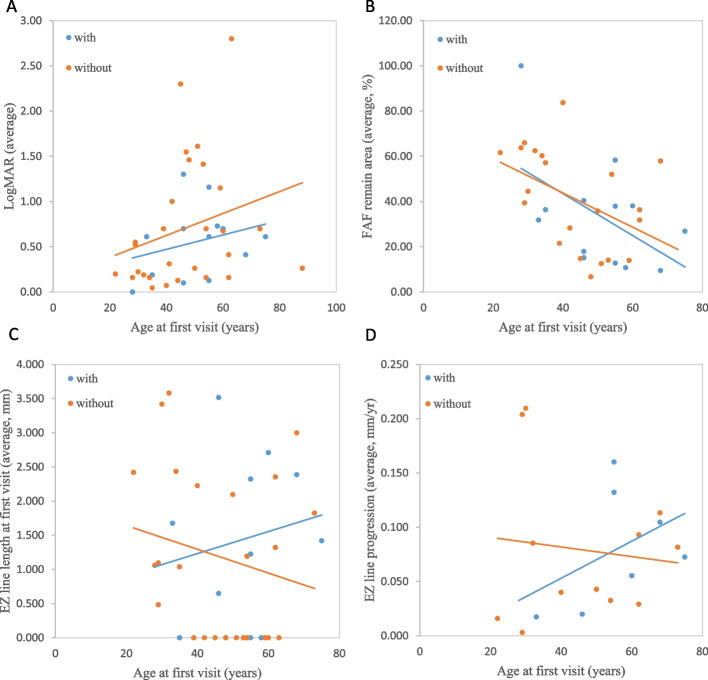
Comparison of the clinical progression of patients with/without c.2802 T > G in our cohort. Every point represents an individual patient. The blue and orange lines are the linear regression lines for those with and without c.2802 T > G, respectively. **A, B** LogMAR of the average of both eyes at the first visit, presented as a function of age. **B** The average intact area of macula of both eyes on fundus autofluorescence (FAF, %) at the first visit, presented as a function of age. **C** EZ line length of the average of both eyes at the first visit, presented as a function of age. **D** EZ line loss progression rates (mm/year) of the average of both eyes, presented as a function of age

Visual acuity (average of both eyes, logMAR) at the first visit was 0.56 in patients with the c.2802T>G variant; whereas, that in those without the c.2802T>G variant was 0.72. Visual acuities (average of both eyes) at a recent visit were 0.72 for patients with the c.2802T>G variant and 0.94 for those without the c.2802T>G variant. Figures 3A showed the average baseline visual acuities of the right and left eyes and both groups showed similar trend.

Figure 3B showed the average preserved areas of the macula at the first visit of both eyes. The average intact areas of the macula at the first visit for those with c.2802T>G variant were 33.24%; while for those without the c.2802T>G variant, the average intact areas were 42.84%. Regarding the progression rate of hypofluorescent areas at the macula, the average rate was 3.95%/year in those with the c.2802T>G variant and 3.69 %/year in those without the c.2802T>G variant.

Figure 3C showed the patients’ average EZ line lengths of both eyes plotted at the first visit. The mean baseline EZ line length was 1.447 mm in patients with the c.2802T>G variant, whereas it measured 1.182 mm in those without the variant. In the analysis of the EZ line loss progression rate at the fovea, the average rates were 0.081 mm/year in patients with the c.2802T>G variant and 0.079 mm/year in those without the c.2802T>G variant. In the linear model showing the progression rate of EZ line loss as a function of age (Fig. 3D), patients with the c.2802T>G variant showed a faster EZ line progression rate along with increasing age.

Because c.2802T>G is a missense mutation, we further analyzed 13 subjects with the c.2802T>G variant to determine the effect of the other allele. Only two (15.4%) had the other allele with a truncated variant, and eleven (84.6%) did not. However, the two subgroups had no significant differences in visual acuity, EZ line length, and remaining FAF areas.

### Clinical presentations of *USH2A*-related retinal dystrophies based on the rate of EZ line progression

Among the 41 patients in our cohort, 19 underwent regular follow-ups with high-quality imaging more than four times. The follow-up period was from 20.43 to 110.10 months, with a mean of 48.34 months. Thirteen of them lost the follow-up of optical coherence tomography (OCT)images, and nine initially showed a destroyed EZ line.

In our cohort, the overall average rate of EZ line progression was 0.082 mm/year in both eyes. To assess the difference based on the rate of EZ line progression, clinical presentations and genotype were compared among the fast progression group (rate > 0.1 mm/year), moderate progression group (rate between 0.05–0.1 mm/year), and slow progression group (rate < 0.05 mm/year), which comprised 6 (32%), 7 (36%), and 6 (32%) patients, respectively.

The age of symptom onset showed the mean value of 32.17, 31.71, and 38.40 years for the fast, moderate, and slow progression subgroups, respectively. Although the age of onset was lower in the fast and moderate progression subgroups than in the slow progression subgroup, the analysis of variance did not show statistical significance.

To further distinguish the difference, we focused on the fast and slow subgroups to analyze the visual acuity and FAF hypofluorescent area progression at the macula. Visual acuities (average of both eyes, logMAR) at the first visit were 0.32 in the slow progression subgroup and 0.34 in the fast progression subgroup, respectively. Visual acuity (average of both eyes) at a recent visit was 0.34 in the slow progression subgroup; whereas, it measured 0.36 in the fast progression subgroup. The average baseline intact areas of the macula of both eyes were 56.94% in the slow progression subgroup and 44.67% in the fast progression subgroup, respectively. However, the difference was not statistically significant.

#### Case presentation

Two patients were selected from groups T- and T+ for a more detailed comparison.

### Case 1

**Fig. 4:**
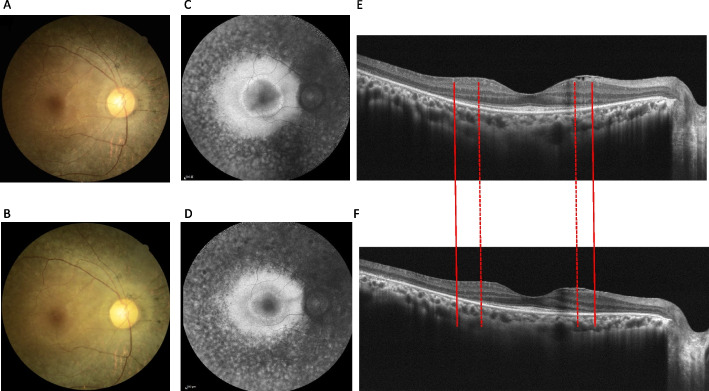
Case presentation from a patient of group T + . Right eye sequential images of color fundus, FAF and optical coherence tomography (OCT) of a 30-year-old woman with one truncated variant and a follow-up period of 37.2 months. **A, B** Color fundus at the first and latest visit. **C, D** FAF showed typical-type RP and a macula hypofluorescent lesion progression rate of 0.813%/year. **E, F** In OCT imaging, the EZ line was intact between the two solid lines at the first visit, whereas the two dotted lines revealed the boundary of the EZ line at a recent visit. The EZ line loss rate at the fovea performed faster, with 0.210 mm/year

The patient (proband 17; 30 years old, with a follow-up period of 37.2 months), who had one truncated variant from group T+, had variants c.5836C>T and c.10859T>C. At the first visit, the patient had an early onset age of 21 years and a visual acuity of 0.22 logMAR for both eyes. Color fundus and FAF images of the right eye at first and recent visits were showed from Fig. 4A-D. FAF images (Fig. 4C and D) showed typical-type RP and a macular hypofluorescent lesion progression rate of 0.813 %/year. OCT imaging (Fig. 4E and F) showed that the EZ line loss rate in the fovea was faster at 0.210 mm/year in the right eye.

### Case 2

**Fig. 5:**
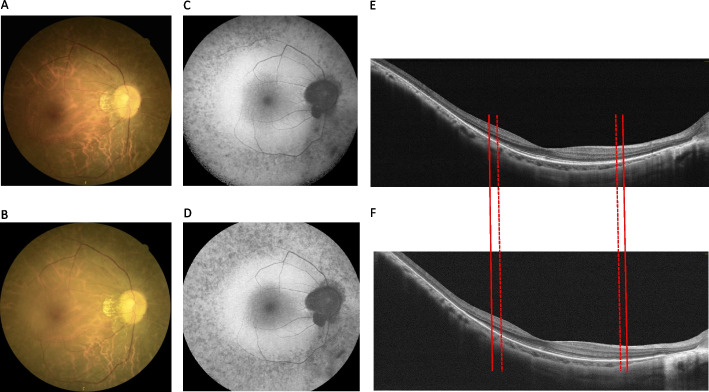
Case presentation from a patient of group T-. Right eye sequential images of color fundus, FAF and optical coherence tomography (OCT) of a 50-year-old woman without truncated variants and with a follow-up period of 37.77 months. **A, B** Color fundus at the first and latest visit. **C, D** FAF showed pericentral-type RP and a macula hypofluorescent lesion progression rate of 2.832%/year. **E, F** In OCT imaging, the EZ line was intact between the two solid lines at the first visit, whereas the two dotted lines revealed the boundary of the EZ line at a recent visit. EZ line loss rate at the fovea performed slower, with 0.020 mm/year

The patient (proband 20; 50 years of age) with no truncated variants from group T- had variants c.2802T>G and c.13007G>A. The patient had a relatively late onset age of 46 years. The mean follow-up period was 37.77 months. Visual acuity at the first visit showed mild impairment with 0.05 logMAR in the right eye and 0.15 logMAR in the left eye. Color fundus and FAF images of the right eye at first and recent visits showed from Fig. 5A-D. FAF images (Fig. 5C and D) showed pericentral-type RP and a macular hypofluorescent lesion progression rate of 2.832 %/year in the right eye. OCT imaging (Fig. 5E and F) showed that the EZ line loss rate at the fovea was slower at 0.020 (mm/year) in the right eye.

## Discussion

We analyzed the genotypes and phenotypes of 41 patients harboring disease-causing variants of the *USH2A* gene. Only a few studies have focused on *USH2A-*associated retinal dystrophies in Taiwan. Chen et al. have reported that *CYP4V2*, *EYS,* and *USH2A* are the three most common disease-causing genes in Taiwanese patients with IRDs. Among these, *USH2A*-related retinal dystrophies was the only potential syndromic disease that had the greatest impact on these patients [21]. Few detailed clinical presentations were described in that study. In this study, we focused on *USH2A*-related retinal dystrophies and followed up on the patients based on visual acuity, FAF, and OCT images. We attempted to quantify the disease progression and evaluate the genotype–phenotype correlation. In addition, among the 82 eyes that had been under investigation, only 1 eye (1.22%) developed lamellar macula hole and 2 eyes (2.44%) developed cystoid macular edema during the follow-up period. Thus, confounding factors such as cataracts, epiretinal membranes and cystoid macular edema were found to have minimal influence in our study.

Approximately 190 mutations in the *USH2A* gene have been reported in patients with USH [22]. In the present study, 46 mutated *USH2A* alleles were identified in 41 Taiwanese patients. Half of them were missense mutations. The patients diagnosed with *USH2A*-related retinal dystrophies in our cohort had a mean age at symptom onset of 31.21 (SD ± 15.24) years. This finding was also consistent with the fact that *USH2A*-related retinal dystrophies are not usually diagnosed until the second decade of life or later in previous studies [23]. Considering that *USH2A*-related retinal dystrophies have a rather late onset, the earlier we can detect this gene in patients, the sooner potential gene therapy can be initiated.

A previous study revealed that mutations in exon 13 are the most common, nearly 18.4% globally, with higher frequencies of 22% in America, 19.2% in Europe, and 12% in East Asia [1]. Our study revealed a prevalence of 21% of exon 13 variants for *USH2A*-related retinal dystrophies in Taiwanese, which is relatively high compared with those in previous studies. In addition, exon 13 variants were mostly composed of c.2802T>G in our cohort. These findings provide great benefits for most patients to receive possible gene therapies for exon 13 in the future [18].

Previous studies have reported that the variants of *USH2A* alleles appear to have geographical or ethnic distributions. c.2299delG was reported to be one of the most frequent variants in *USH2A* patients in Europe and the United States, which accounts for 47.5% in Denmark, 36% in Scandinavia [24], and 31% in the Netherlands [7]. However, c.2299delG has never been detected in Chinese patients, and our study did not find it either. The most frequently detected variants in the Chinese patients were c.2802T>G and c.8559-2A>G [12]. In our cohort, c.2802T>G was the most predominant, with a relative allele frequency of 15.85%. Moreover, only one patient was harboring c.8559-2A>G, with a relative allele frequency of 1.22%. c.4576G>A was the second most common variant in our study, with a relative allele frequency of 7.32%. Hence, we suggest that there might be a founder effect in the Taiwanese population.

In the current study, we compared the clinical characteristics between the two groups: group T- that did not have truncated variants and group T+ that had at least one truncated variant. Based on our findings, truncated alleles appear to be related to disease severity. The results revealed that the age at symptom onset and the first visit were significantly lower in subjects with at least one truncated variant. This is consistent with the results of previous studies [25, 26]. The visual acuities at the first and recent visits were worse in patients with truncated alleles than in those without. Although no statistically significant differences were identified, similar studies have reported worse visual acuity in patients with more severe *USH2A* variants [27].

We analyzed structural data using the EZ line length at the fovea via SD-OCT, as it has become one of the most important tools for evaluating macular involvement in IRDs. The EZ hyper-reflective line represents the photoreceptor inner/outer segment junction, and its measurement can correlate with functional data such as visual acuity and field [28, 29]. In our study, the baseline EZ line length was shorter and the EZ line loss progression rate (mm/year) was faster in group T+ than in group T-, yet both of them did not reach statistical significance. We presumed that these results were due to the small sample size of our database.

We searched for FAF imaging studies of RP or USH, most of which compared the difference or existence of a hyperautofluorescent ring at the macula [12]. Therefore, we introduced a sequential variable, the hypofluorescent area progression rate at the macula (%/year). In our study, the baseline intact macular area was larger in patients with truncated variants than in those without, although the difference was not significant. This could be attributed to the younger population in group T+ with a better-preserved macular region. Additionally, it is noteworthy that *USH2A*-related retinal dystrophies often initiate retinal degeneration from the peripheral retina, resulting in macular involvement occurring later in life.

In addition, hearing loss was significantly higher in the group T+. This suggested that truncated alleles might lead to hearing impairment and severe disease progression.

c.2802T>G (p.Cys934Trp) is a missense variant with deleterious effects. Lenassi et al. defined c.2802T>G into the group of “retinal disease-specific” alleles mainly associated with diseases confined to the eye. Ten patients (10/13, 76.9%) in our cohort presented normal hearing. This is compatible with the fact that at least one “retinal disease-specific” *USH2A* allele in a patient with *USH2A-*related disease led to the preservation of normal hearing. In the present study, other clinical presentations were also analyzed. Similar results were observed for the c.2802T>G variant. Moreover, among the 13 subjects who were harboring the c.2802T>G variant, only two of them (15.4%) were put into group T+ and eleven (84.6%) of them in group T-. The results also revealed no significant differences in visual acuity, EZ line length, and FAF remaining areas between the two subgroups. Thus, we suggest that the influence of the c.2802T>G variant on clinical presentation is inconclusive and could be affected by other features. Further studies are required to determine whether the c.2802T>G variant results in milder disease progression.

Several previous studies have analyzed EZ line progression in patients with RP, including those conducted in populations with arRP, autosomal dominant (adRP), and X-linked RP (XLRP) [30–32]. Furthermore, they suggested that adRP was the mildest form of RP and XLRP was the most severe. When stratifying the patients, the progression rates showed: −0.095 mm/year for adRP, −0.128 mm/year for arRP, and −0.219 µm mm/year for XLRP. We focused on *USH2A*-related retinal dystrophies, a form of arRP. In our study, the average progression rates were −0.078 mm/year in the right eye and −0.081 mm/year in the left eye and were lower than that of the aforementioned cohort. We presumed that this might be related to the small sample size in our study (*n* = 19) and the different disease statuses in each patient. Additionally, we classified the progression rates of the EZ line into three groups. Nevertheless, we could not observe a significant difference in visual acuity at the first/recent visit or in the hypofluorescent area progression rate at the macula (%/year) among the groups with different EZ line loss rates. This could be attributed to the limitation of using the EZ line because it cannot be used in the later stages of the disease. In late RP, the outer retina is nearly atrophic and the EZ line is not easily detected; however, the hypofluorescent area in the FAF image still remains measurable. We suggest that the structural variables of the EZ line and FAF hypofluorescence area should both be assessed in patients with RP; nevertheless, the EZ line variables might be more sensitive in the early stage, and the FAF hypofluorescence area might be more useful during the advanced stage of the disease.

### Limitations of this study

The present study has certain limitations: (i) as this was a retrospective study, missing data may have led to biased results; (ii) as patients were presented at different stages of the disease, a small number of patients were already in the end stage of the disease, and data such as EZ line length could not be obtained or classified; (iii) as *USH2A*-related retinal dystrophies are relatively rare diseases, the sample size may not have been sufficiently large to yield statistically significant results and was susceptible to the influence of outliers; (iv) patients with hearing loss tended to be more sensitive to visual function tests because of their greater dependence on visual functions in daily life. These patients may have undergone ophthalmological examination at a younger age and thereby may have received an earlier diagnosis, which accounts for a selection bias; and (v) we did not perform segregation analysis on all of our patients. Whether the genetic test was done or not depended on patients' willingness since the genetic test is not covered by our health insurance and they had to pay by themselves for each sample. Future studies with larger sample sizes and longer follow-up periods may provide more definitive insights into this disease.

## Conclusions

This is the first cohort study to provide detailed genetic and longitudinal clinical analyses of patients with *USH2A-*related retinal dystrophies in Taiwan. The mutated allele frequency in exon 13 was high in Taiwan due to the predominant c.2802T>G variant. Moreover, truncated variants greatly impacted disease progression and could determine the length of therapeutic windows. These findings provide insight into the characteristics of candidates for future gene therapies.

### Methods

#### Subjects and clinical evaluation

The patients included in the present study were recruited as part of Taiwan inherited retinal degeneration project (TIP), which was approved by the Research Ethics Committee of National Taiwan University Hospital (IRB No.:201408082RINC). Between September 2015 and December 2022, 41 patients with *USH2A*-related retinal dystrophies were recruited from 39 families. The participants recruited in our program underwent a series of comprehensive ophthalmic examinations, including visual acuity, color fundus photography, OCT, and FAF at the Department of Ophthalmology, National Taiwan University Hospital, Visual acuity was determined using the Snellen chart, and the results were converted to the logMAR scale for analysis. The visual acuity measures “hand movement,” “light perception,” and “no light perception” that were assigned logMAR values of 2.3, 2.8, and 2.9, respectively. In our study, hearing loss was defined as patients who could not hear normal conversations at the clinical visit, which is generally accepted as 60 dB [33]. The definite diagnosis for each subject was established based on the above examinations, clinical presentations, genetic analysis, and family history. Symptom onset was referred specially as the age when the patients reported nyctalopia and/or visual field constriction.

#### Next-generation sequencing (NGS)

Blood samples were collected after obtaining informed consent. Genomic DNA was extracted from the peripheral blood leukocytes using a DNA extraction kit (Gentra Puregene Blood Kit; QIAGEN, Hilden, Mettmann, Germany). Genetic testing was performed using a probe capture-based NGS approach targeting 212 IRD-associated genes, which were selected from the RetNet (https://sph.uth.edu/retnet/) and OMIM (https://www.ncbi.nlm.nih.gov/omim) databases (Additional file 1).

#### EZ Line measurement with OCT

A Spectralis HRA + OCT device (Heidelberg Engineering, Heidelberg, Germany) with an 870 nm light source and an automatic real-time registration program were used to collect the OCT images. The EZ line was measured manually on horizontal scans taken through the fovea using Spectralis software. The EZ line progression rate was obtained by dividing the difference in EZ line length at the first and recent visit by the total follow-up period of each patient.

#### Lesion area measurement with FAF

To determine the degree of disease progression in the macula, we analyzed FAF images using ImageJ (Version 1.51). For the area of interest, which was a circle in our study, we first set the center of the circle at the foveola, and the radius was assigned as the distance from the foveola to the center of the optic disc. We measured the hypofluorescent area by adjusting the brightness of the color threshold. The value of interest was obtained as the percentage (%) of the area of hypofluorescence in the hypothetical circle divided by the area of the hypothetical circle for the correction of different background brightness in each FAF. The still intact area (%) was obtained by subtracting the hypofluorescent area (%) from 100. The hypofluorescence progression rate was obtained by dividing the difference in the hypofluorescent area at the first and recent visit by the total follow-up period of each patient.

### Classification system

#### Genotype grouping based on the existence of truncated alleles

To assess the influence of the variant location on clinical presentation, the patients were classified into two groups: group T- patients, who had no truncated variants, and group T+ patients, who had at least one variant with truncated mutations, which are predicted to result in nonsense-mediated decay or significant truncation of the protein product.

### Genotype grouping based on the existence of c.2802T>G variant

According to previous studies, the c.2802T>G mutation could be detected in East Asian populations, with an alternative allele frequency of 0.004 in the 1000 Genomes Project East Asian database and 0.00279 in the Exome Aggregation Consortium East Asian database [34]. In our cohort, c.2802T>G (p.Cys934Trp) was the most predominant variant, accounting for 15.85% (13/82), and were all heterogeneous types. Patients were divided into two groups: patients with at least one allele of the c.2802T>G variant and patients who had no c.2802T>G variant.

#### Phenotype grouping based on EZ line progression

To investigate the correlation between the rate of EZ line progression and the clinical presentation, the subjects with data on EZ line progression were divided into three groups based on the average of both eyes: patients with a progression rate over 0.1 mm/year, patients with a progression rate between 0.05 and 0.1 mm/year, and patients a progression rate lower than 0.05 mm/year.

#### Statistical analysis

The results are presented as the mean ± standard error of the mean with a 95% confidence interval. Student’s t-test, analysis of variance, chi-square test, and Pearson’s correlation, followed by post-hoc multiple comparisons, were performed using SPSS (version 27.0; SPSS Inc., Chicago, IL, USA) and Excel (Microsoft 365).

### Supplementary Information

Additional file 1. Known genes associated with inherited retinal dystrophies (*N* = 212).

## Data Availability

The sequencing raw data (FASTQ files) analyzed during the current study available in the NCBI Sequence Read Archive (SRA) (PRJNA1037691). The data of non-sequencing data and materials are available on reasonable request from the corresponding authors.
