# Supplementary material for: High prevalence of exon-13 variants in USH2A-related retinal dystrophies in Taiwanese population
Source: Orphanet J Rare Dis. 2024 Jun 15;19:238. doi: 10.1186/s13023-024-03238-2 (PMC11179209; doi:10.1186/s13023-024-03238-2)
Supplement: Supplementary file 1 — Additional file 1. Known genes associated with inherited retinal dystrophies (N = 212). [file 13023_2024_3238_MOESM1_ESM.docx]

| **Additional file 1. Known genes associated with inherited retinal dystrophies (N = 212).** | |
| --- | --- |
| **Disease category** | **Genes** |
| Bardet-Biedl syndrome, AR | *ARL6, BBIP1, BBS1, BBS2, BBS4, BBS5, BBS7, BBS9, BBS10, BBS12,C8orf37,CEP290, IFT172, IFT27, INPP5E, KCNJ13, LZTFL1, MKKS, NPHP1, SDCCAG8, TRIM32, TTC8* |
| Chorioretinal atrophy or degeneration, AD | *PRDM13, RGR* |
| Cone or cone-rod dystrophy, AD | *AIPL1, CRX, GUCA1A, GUCY2D, PITPNM3, PROM1, PRPH2,*  *RIMS1, SEMA4A, UNC119* |
| Cone or cone-rod dystrophy, AR | *ABCA4, ADAM9, ATF6, C21orf2, C8orf37, CACNA2D4, CDHR1, CERKL, CNGA3, CNGB3, CNNM4, GNAT2, KCNV2, PDE6C, PDE6H, POC1B, RAB28, RAX2, RDH5, RPGRIP1, TTLL5* |
| Cone or cone-rod dystrophy, XL | *CACNA1F, RPGR* |
| Congenital stationary night blindness, AD | *GNAT1, PDE6B, RHO* |
| Congenital stationary night blindness, AR | *CABP4, GNAT1, GNB3, GPR179, GRK1, GRM6, LRIT3, RDH5, SAG, SLC24A1, TRPM1* |
| Congenital stationary night blindness, XL | *CACNA1F, NYX* |
| Deafness alone or syndromic, AD | *WFS1* |
| Deafness alone or syndromic, AR | *CDH23, CIB2, DFNB31, MYO7A, PCDH15, USH1C* |
| Leber congenital amaurosis, AD | *CRX, IMPDH1, OTX2* |
| Leber congenital amaurosis, AR | *AIPL1, CABP4, CEP290, CRB1, CRX, DTHD1, GDF6, GUCY2D,*  *IQCB1, KCNJ13, LCA5, LRAT, NMNAT1, PRPH2, RD3, RDH12,*  *RPE65, RPGRIP1, SPATA7, TULP1* |
| Macular degeneration, AD | *BEST1, C1QTNF5, ELOVL4, FSCN2, GUCA1B, HMCN1, IMPG1,*  *OTX2, PRDM13, PROM1, PRPH2, RP1L1, TIMP3* |
| Macular degeneration, AR | *ABCA4, DRAM2, IMPG1* |
| Macular degeneration, XL | *RPGR* |
| Age-related macular degeneration (AMD) | *ABCA4 FBLN5, HMCN1, RAX2* |
| Retinitis pigmentosa, AD | *BEST1, CA4, CRX, FSCN2, GUCA1B, HK1, IMPDH1, IMPG1,*  *KLHL7, NR2E3, NRL, PRPF3, PRPF4, PRPF6, PRPF8, PRPF31,*  *PRPH2, RDH12, RHO, ROM1, RP1, RP9, RPE65, SAG, SEMA4A,*  *SNRNP200, TOPORS* |
| Retinitis pigmentosa, AR | *ABCA4, AGBL5, ARL6, ARL2BP, BBS1, BBS2, BEST1, C2orf71,*  *C8orf37, CERKL, CLRN1, CNGA1, CNGB1, CRB1, CYP4V2,*  *DHDDS, DHX38, EMC1, EYS, FAM161A, HGSNAT, IDH3B, IFT172, IMPG2, KIAA1549, KIZ, LRAT, MAK, MERTK, MVK, NEK2, NEUROD1, NR2E3, NRL, PDE6A, PDE6B, PDE6G, PRCD, PROM1,*  *RBP3, RGR, RHO, RLBP1, RP1, RP1L1, RPE65, SAG, SAMD11,*  *SLC7A14, SPATA7, TTC8, TULP1, USH2A, ZNF408, ZNF513* |
| Retinitis pigmentosa, XL | *OFD1, RP2, RPGR* |
| Retinoschisis, XL | *RS1* |
| Syndromic/systemic diseases with retinopathy, AD | *ABCC6, ATXN7, COL11A1, COL2A1, JAG1, KCNJ13* |
| Syndromic/systemic diseases with retinopathy, AR | *ABCC6, ABHD12, ACBD5, ADAMTS18, AHI1, ALMS1, CC2D2A, CEP290, COL9A1, CSPP1, ELOVL4, FLVCR1, GNPTG, HARS,*  *HGSNAT, INPP5E, INVS, IQCB1, LAMA1, LRP5, NPHP1,*  *NPHP3, NPHP4, PANK2, PCYT1A, PEX1, PEX2, PEX7, PHYH,*  *PNPLA6, POC1B, PRPS1, RDH11, RPGRIP1L, SDCCAG8,*  *TMEM216, TMEM237, TTPA, TUB, WDPCP, WFS1, ZNF423* |
| Syndromic/systemic diseases with retinopathy, XL | *OFD1* |
| Usher syndrome, AR | *ABHD12, CDH23, CEP250, CIB2, CLRN1, DFNB31, HARS, MYO7A, PCDH15, USH1C, USH1G, USH2A* |
| Other retinopathy, AD | *BEST1, CRB1, FZD4, ITM2B, LRP5, MAPKAPK3, MIR204, RCBTB1, TSPAN12, ZNF408* |
| Other retinopathy, AR | *ASRGL1, BEST1, CDH3, CNGA3, CNGB3, CNNM4, CYP4V2,*  *LRP5, MVK, NR2E3, OAT, PROM1, RCBTB1, RLBP1* |
| Other retinopathy, XL | *CACNA1F, CHM, NDP, OPN1LW, OPN1MW, PGK1, RS1* |
| Other putative genes | *ADGRA3, ARL13B, CLRN3, COL11A2, GPR143, OR2W3, PEX26, TMEM67* |
| Macular degeneration, AD | *BEST1, C1QTNF5, ELOVL4, FSCN2, GUCA1B, HMCN1, IMPG1,*  *OTX2, PRDM13, PROM1, PRPH2, RP1L1, TIMP3* |
| Data source from RetNet (<https://sph.uth.edu/retnet/>). AD, autosomal dominant; AR, autosomal recessive; XL, X-linked | |
